# Construct validity and reliability of the Dementia Test for People with Intellectual Disability: neuropsychological test battery for assessing cognitive functioning in people with intellectual disability

**DOI:** 10.1192/bjo.2024.847

**Published:** 2025-03-24

**Authors:** Tanja Sappok, Miriam Flachsmeyer, Peggy Rösner, Björn Kruse, Sandra-Verena Müller, Daria Tarasova

**Affiliations:** Medical School and University Medical Center OWL, Mara Hospital, University Clinic for People with Neurodevelopmental Disorders, Bielefeld University, Bielefeld, Germany; Medical Faculty, Charité University, Berlin, Germany; Department for Neurodevelopmental Disorders, Evangelisches Krankenhaus Königin Elisabeth Herzberge, Berlin, Germany; Faculty for Social Work, Ostfalia University of Applied Science, Wolfenbüttel, Germany

**Keywords:** Dementias/neurodegenerative diseases, diagnostic medicine, intellectual disability, longitudinal data, psychometrics

## Abstract

**Background:**

People with an intellectual disability are vulnerable to additional disorders such as dementia. Psychometrically sound and specific instruments are needed for assessment of cognitive functioning in cases of suspected dementia.

**Aims:**

To evaluate the construct and item validity, internal consistency and test–retest reliability of a new neuropsychological test battery, the Dementia Test for People with Intellectual Disability (DTIM).

**Method:**

The DTIM was applied to 107 individuals with intellectual disability with (*n* = 16) and without (*n* = 91) dementia. The psychometric properties of the DTIM were assessed in a prospective study. The assessors were blinded to the diagnostic assignment.

**Results:**

Confirmatory factor analysis at the scale level showed that a one-factor model fitted the data well (root mean square error of approximation < 0.06, standardised root mean square residual < 0.08, comparative fit index > 0.9). At the domain level, one-factor models showed reasonable-to-good fit index for five of seven domains. Internal consistency indicated excellent reliability of the overall scale (Cronbach’s α: 0.94 for dementia and 0.95 for controls). Item analysis revealed a wide range of difficulties (0.19–0.75 for dementia, 0.31–0.87 for controls), with minimal floor and ceiling effects. Eleven items (26%) had discrimination value ≤ 0.50. Test–retest reliability (*n* = 82) was high, with intraclass correlations of 0.95 (total score) and 0.69–0.96 (domains).

**Conclusions:**

The DTIM fits a one-factor model and demonstrates internal and test–retest reliability; thus, it is suitable for use in cases of suspected dementia in people with various intellectual disabilities.

Owing to increased life expectancy, people with an intellectual disability are increasingly developing age-associated diseases such as dementia.^1^ Dementias occur earlier in such people than in the general population, and their risk of dementia is five times that of people without cognitive impairments. No differences in prevalence rates have been observed across various levels of severity of intellectual impairment.^2^ Aetiologically, Alzheimer’s-type dementia is most commonly found in individuals with an intellectual disability, although dementia with cerebrovascular causes, Lewy body disease, frontotemporal dementia and other forms may also occur.^3^ In particular, people with Down syndrome are more likely to develop Alzheimer disease.^3^

## Challenges in diagnosing dementia in people with intellectual disabilities

Diagnostic clarification of suspected dementia is central to guideline-based treatment. However, this is challenging, because early symptoms can be subtle and may be masked by lifelong cognitive impairments. The challenge is to differentiate between new-onset cognitive deficits and premorbid existing impairments of intellectual performance and adaptive behaviour. Owing to variations in premorbid levels of functioning from person to person, the ‘typical’ primary cognitive deficits (learning and memory problems) may not be noticed, and secondary symptoms such as disorientation, social withdrawal and changes in social behaviour, or sleep disorders may indicate the cognitive decline. A careful diagnosis of dementia, however, is essential for people with an intellectual disability, because it is crucial for adapted and comprehensive treatment and support.^3^ Diagnosis is challenging, especially with this group of people.^4^ So far, there has been a lack of guidelines for diagnosis of dementia in people with an intellectual disability.

The diagnostic process comprises a detailed anamnesis including previous and current diseases and medicinal treatments and a thorough physical examination. In addition to the clinical–neurological examination, cerebral imaging, blood tests and cerebrospinal fluid diagnostics should be carried out. It is important to rule out treatable causes of dementia such as thyroid dysfunction (e.g. autoimmune thyroiditis), hypoparathyroidism, vitamin deficiencies, cerebral tumours or infections.^5^ Standardised neuropsychological testing is recommended to increase diagnostic certainty.^6^

## Necessity for standardised neurocognitive assessment

A special feature of dementia diagnostics in people with an intellectual disability is the need for repeated neuropsychological testing at intervals.^1,5,6^ Currently, the strong heterogeneity of cognitive abilities does not allow comparison with values of the general population or of people with an intellectual disability. The results of neurocognitive assessments are therefore compared with the individual baseline level of each person.^7^ For this reason, dementia can usually only be diagnosed as it progresses.

For assessment of neurocognitive decline, standardised normed tests such as the CERAD (Consortium to Establish a Registry for Alzheimer’s Disease) test battery are used in the general population. In people with an intellectual disability, the assessment can use the neuropsychological test battery by Burt and Aylward,^8^ which has also been validated in German.^5^ Specifically for people with Down syndrome, the Cambridge Examination for Mental Disorders of Older People with Down’s Syndrome and Others with Intellectual Disabilities (CAMDEX-DS)^9^ has been developed and validated in German.^10^ However, to the best of our knowledge, there is a lack of neuropsychological test batteries for people with an intellectual disability, specifically, for people without Down syndrome.

## Additional informant-based assessment

In addition to neurocognitive assessments, an informant-based assessment of daily practical skills and adaptive behaviour is supportive of the diagnostic process.^11,12^ Third-party observation procedures frequently used with this group of people include the Checklist for the Assessment of Dementia in People with Intellectual Disabilities,^13^ the Dementia Questionnaire for People with Learning Disabilities^14^ and the Dementia Screening Questionnaire for individuals with intellectual disabilities (DSQIID).^11^ The DSQIID is easy to use in clinical practice and has already been integrated into the National Task Group – Early Detection Screen for Dementia in 2013.^15,16^ Startin et al^17^ developed a third-party observation procedure that focused on cognitive abilities in people with trisomy 21, the Cognitive Scale for Down Syndrome (CS-DS).^17,18^

## The Dementia Test for People with Intellectual Disability

In summary, various neuropsychological test procedures have been developed specifically for people with an intellectual disability; however, few of these have been assessed with respect to reliability and validity, especially in various languages.^6,16^ Moreover, they may have been developed only for certain subgroups, such as the CAMDEX-DS. To address the need for a standardised, psychometrically sound neuropsychological assessment for people with intellectual disabilities in German, Müller and Kuske constructed the Dementia Test for People with Intellectual Disability (DTIM).^19^ The guiding principle in the development of the DTIM was the need to develop an instrument that could be used with a heterogeneous group with varying degrees of cognitive impairment. For this reason, the DTIM does not focus on a single aetiology but explicitly considers people with more severe disabilities to ensure broad applicability. In addition, graduated aid is possible in the application of the scale. The DTIM is a direct neuropsychological examination that should be used in combination with the authorised German translation of the DSQIID as a third-party observation questionnaire. The DTIM is used to evaluate a person’s current mental status: people with higher levels of cognitive functioning are expected to achieve higher DTIM scores. As the premorbid state of cognitive functioning depends on the severity of intellectual disability, repeated assessments are necessary to observe individual changes over time. A decline in DTIM scores in between two different points of time provides evidence of a decline in cognitive functioning, supporting the dementia diagnosis. The construct ‘cognitive functioning’ includes several cognitive processes that are typically assessed during a mental status examination in patients with suspected dementia.^20^ Specifically, the DTIM assesses the following cognitive domains: orientation, language, attention, memory, planning, abstract logical thinking and visual perception. Similar functions of neurocognition are examined using standardised instruments such as the CAMDEX-DS.

## Study aim

The aim of this study was to investigate the psychometric properties of the DTIM to develop a valid and reliable instrument for structured neuropsychological assessment of individuals with intellectual disability and suspected dementia. We examined the construct validity at the overall and domain levels, as well as item validity and reliability in terms of internal consistency and test–retest reliability; test validity was not the primary outcome.

## Method

### Study design

The study was performed in an out-patient clinic responsible for psychiatric treatment and care for people with an intellectual disability in a metropolitan area of Germany. Inclusion criteria were (a) age above 40 years for people with an intellectual disability due to Down syndrome or above 55 years for people with an intellectual disability of other aetiologies, and (b) diagnosis or suspicion of dementia; exclusion criteria were profound levels of intellectual disability, lack of consent, or a severe physical or sensory impairment which made it impossible to take part in the assessment. Independent, multi-professional case conferences were conducted to confirm or rule out the dementia diagnosis. This reference diagnosis was based on a comprehensive anamnestic, clinical and standardised neuropsychological work-up with the scale of Burt and Aylward as described in Rösner et al.^5^

**Fig. 1:**
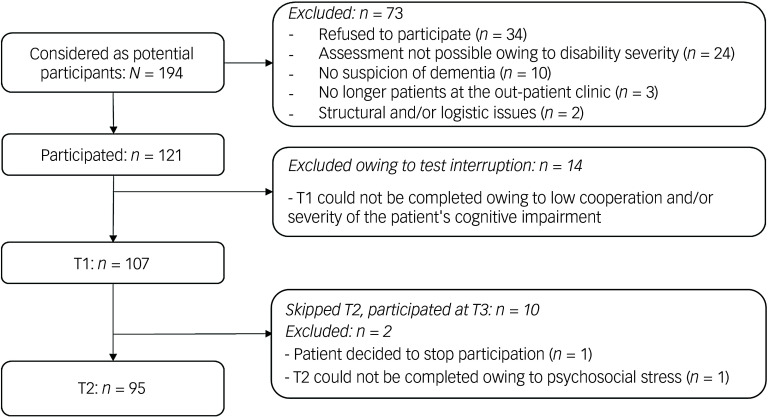
Recruitment process, showing numbers of participants at different timepoints (T1, baseline; T2, 6 months afterwards).

The DTIM assessments were conducted by trained neuropsychologists who were blind to the results of the case conferences. For the current analysis, the DTIM was applied at two different time points: baseline (T1) and 6 months (T2) afterwards. Figure 1 shows a detailed description of the recruitment process up to T2. The results of the baseline assessment (T1) were used for item, reliability and construct validity analysis. Test–retest reliability was examined in the control group (without dementia) by correlating the results at T1 with those of the second assessment after 6 months (T2). Baseline variables, such as demographic information (e.g. age and sex) and information on medication, additional disorders and further medical investigations, were systematically recorded upon the first assessment.

### Ethics statement

The authors assert that all procedures contributing to this work comply with the ethical standards of the relevant national and institutional committees on human experimentation and with the Helsinki Declaration of 1975, as revised in 2013. Ethical approval was obtained by the ethics committee of the Königin Elisabeth Herzberge Hospital in Berlin (5 April 2017). The ethics committee of the Ostfalia University of Applied Science Wolfenbüttel also approved this study (28 June 2017; resolution number 2/2017). Written informed consent for participation was given by the participants or their legal guardians. Consent forms and information about the study were available in German and easy-to-use German.

### Study population

A sample of 107 adults with intellectual disability was recruited between February 2018 and June 2021; 15% of these were diagnosed with dementia, and 52% were female (Table 1). Mild, moderate and severe levels of intellectual disability were present. The mean age was 54 years. Down syndrome was observed in 40 individuals. Of the 107 participants, 95 took part in the second assessment after 6 months; drop-out was mainly for logistic reasons. Eighty-two participants at T2 received no dementia diagnosis, and 13 were diagnosed with dementia. Affective disorders were more often diagnosed in people without dementia.

**Table 1:** Sample characteristics at baseline

Characteristic	Dementia (*n* = 16)	No dementia (*n* = 91)	*P*-value
Gender (female), *n* (%)	11 (69)	45 (49)	0.25^a^
Severity of intellectual disability, *n* (%)			0.72^a^
Mild	3 (19)	23 (25)	
Moderate	10 (62)	47 (52)	
Severe	3 (19)	21 (23)	
Psychiatric disorders			
F06, *n* (%)	6 (38)	21 (23)	0.22^a^
F1, *n* (%)	1 (6)	5 (5)	1.00^a^
F2, *n* (%)	0 (0)	22 (24)	0.06^a^
F3, *n* (%)	2 (0)	52 (57)	**0.003**^a^
F4, *n* (%)	0 (0)	12 (13)	0.27^a^
F8, *n* (%)	0 (0)	19 (21)	0.10^a^
Age in years, mean (s.d.)	54.94 (5.88)	53.44 (11.71)	0.60^b^
Challenging behaviour, mean (s.d.)	21.18 (16.73)	31.34 (24.21)	0.18^b^
Psychoactive medication, *n* (%)	13 (81)	79 (87)	0.84^a^
Down syndrome, *n* (%)	11 (69)	29 (32)	**0.01**

a . Chi-squared test.

b . Wilcoxon rank-sum test; significant results are marked in bold. Behaviour is defined according to the Royal College of Psychiatrists (2007) as challenging when ‘it is of such an intensity, frequency or duration as to threaten the quality of life and/or the physical safety of the individual or others and is likely to lead to responses that are restrictive, aversive or result in exclusion’.^21^

### Dementia Test for People with Intellectual Disability

The DTIM is an interactive neuropsychological assessment administered by a trained clinician. A familiar caregiver should be present to give the participant a sense of security and provide emotional support.^19^ The neuropsychological test battery of the DTIM contains 43 items in 7 domains, i.e. orientation, language, attention, memory, planning, abstract logical thinking and visual perception. Table 2 contains examples of items in each domain. Some tasks within the DTIM progressively increase in difficulty, aiming to minimise ceiling and floor effects.^19^ For each task, a participant can score 1 to 6 points (the maximum attainable score varies between tasks). The score is determined according to the instructions (whether the task was completed correctly or partly correctly, whether additional cues were needed, etc.). The results within the different domains are summed, with a maximum score of 76 points (Table 3). At each assessment point, the participants’ current state of cognitive functioning is assessed based on their performance. The T1 results represent an individual’s baseline and serve as a reference point for future assessments. In cases of dementia, cognitive decline can be observed over time, providing crucial information for the diagnosis. In the absence of reliable norm values, the result of the first assessment represents an individual point of reference for further comparison. An individual baseline is calculated for each subject. This takes into account the fact that some individuals do not have the skills to perform the tasks owing to their disability. Thus, everyone becomes their own reference. A decline in neuropsychological functioning indicates the presence of dementia. No clear cut-off score has been defined so far. On the basis of their own clinical experience with application of the DTIM, the test developers recommend a decline of 5 points in the overall score.^22^ Administration of the test takes about 60 min.

**Table 2:** Examples for Dementia Test for People with Intellectual Disability items in each domain

Domain	Exemplary items
Orientation	The participants are asked to recall their full name (item 1.1) and age (item 1.2)
Language	The examiner shows a pen to the participants and asks them to name it (item 2.4) The examiner asks the participants to name as many animals as possible within 1 min (item 2.6)
Attention	The examiner puts ten pens in front of the participants and asks them to count them aloud (item 3.4) The examiner reads number sequences of increasing length and asks the participants to repeat them (item 3.5)
Memory	The examiner puts three pictures in front of the participants and asks them to name the objects on the pictures. Immediately after that, the pictures are turned around, and the participants are requested to recall the objects (item 4.1)
Planning	The participants are requested to follow a row of simple commands: touch their own shoulder, clap their hands and put their hands on the table (item 5.5)
Abstract logical thinking	The examiner presents a pattern with a missing part. The participants are asked to complete the presented sequence by selecting a part from several given options (items 6.3–6.5)
Visual perception	The participants are requested to copy figures presented on a worksheet (items 7.1–7.3)

**Table 3:** Item analysis: item difficulty, standard deviation and discrimination power

Item (maximum points)	Item difficulty		Standard deviation		Discrimination	
	Controls	Dementia	Controls	Dementia	Controls	Dementia
1. Orientation						
1.1 (max. 2)	0.87	0.75	0.60	0.82	0.50	0.60
1.2 (max. 1)	0.42	0.19	0.50	0.40	0.61	0.43
1.3 (max. 1)	0.42	0.25	0.50	0.45	0.69	0.58
1.4 (max. 1)	0.44	0.44	0.50	0.51	0.65	0.63
1.5 (max. 1)	0.57	0.44	0.50	0.51	0.66	0.39
1.6 (max. 1)	0.65	0.31	0.48	0.48	0.55	0.23
1.7 (max. 1)	0.31	0.25	0.46	0.45	0.71	0.65
1.8 (max. 1)	0.51	0.31	0.50	0.48	0.74	0.60
2. Language						
2.1 (max. 3)	0.49	0.35	10.42	10.30	0.68	0.68
2.2 (max. 1)	0.26	0.19	0.44	0.40	0.57	0.48
2.3 (max. 1)	0.86	0.81	0.35	0.40	0.56	0.60
2.4 (max. 1)	0.84	0.81	0.37	0.40	0.56	0.60
2.5 (max. 3)	0.54	0.44	10.35	10.40	0.71	0.73
2.6 (max. 3)	0.70	0.42	10.14	10.30	0.76	0.69
3. Attention						
3.1 (max. 1)	0.98	0.94	0.15	0.25	0.07	0.47
3.2 (max. 1)	0.81	0.75	0.39	0.45	0.16	0.35
3.3 (max. 1)	0.71	0.50	0.45	0.52	0.48	0.34
3.4 (max. 3)	0.74	0.67	10.24	10.32	0.49	0.72
3.5 (max. 3)	0.45	0.31	0.95	0.85	0.60	0.62
4. Memory						
4.1 (max. 3)	0.70	0.27	10.19	10.11	0.76	0.71
4.2 (max. 6)	0.68	0.10	20.54	10.63	0.78	0.68
4.3 (max. 3)	0.67	0.31	10.23	10.06	0.73	0.73
4.4 (max. 1)	0.60	0.44	0.49	0.51	0.53	0.73
5. Planning						
5.1 (max. 1)	0.99	0.88	0.10	0.34	0.28	0.03
5.2 (max. 1)	0.95	10.00	0.23	0.00	0.34	
5.3 (max. 1)	0.77	0.69	0.42	0.48	0.61	0.02
5.4 (max. 2)	0.62	0.50	0.78	0.82	0.67	0.86
5.5 (max. 3)	0.65	0.37	10.07	0.89	0.70	0.60
5.6 (max. 4)	0.87	0.75	0.99	10.21	0.73	0.55
5.7 (max. 6)	0.72	0.58	20.17	20.48	0.70	0.77
5.8 (max. 1)	0.82	0.50	0.38	0.52	0.58	0.68
5.9 (max. 1)	0.86	0.56	0.35	0.51	0.55	0.62
6. Abstract logical thinking						
6.1 (max. 1)	0.69	0.50	0.46	0.52	0.39	0.27
6.2 (max. 2)	0.48	0.41	0.89	0.98	0.51	0.23
6.3 (max. 1)	0.46	0.31	0.50	0.48	0.51	0.38
6.4 (max. 1)	0.35	0.06	0.48	0.25	0.51	0.59
6.5 (max. 1)	0.14	0.06	0.35	0.25	0.39	0.59
7. Visual perception						
7.1 (max. 1)	0.74	0.69	0.44	0.48	0.63	0.72
7.2 (max. 1)	0.62	0.44	0.49	0.51	0.75	0.77
7.3 (max. 2)	0.38	0.28	0.83	0.73	0.60	0.68
7.4 (max. 1)	0.69	0.69	0.46	0.48	0.61	0.36
7.5 (max. 1)	0.68	0.50	0.47	0.52	0.40	0.51
7.6 (max. 1)	0.79	0.56	0.41	0.51	0.58	0.78

### Reference criteria

Diagnosis of dementia was based on the ICD-10 diagnostic criteria in a consensus conference.^23^ The clinical assessment included a thorough medical history, a neurological and psychiatric examination, laboratory diagnostics and brain imaging, as well as standardised neuropsychological testing with the neurological test battery by Burt and Aylward^8^ and the Dementia Questionnaire for People with Learning Disabilities.^14^ The diagnostic decision for or against dementia was made in a multi-professional case conference. The professionals involved in the case conferences were blind to the DTIM results. Likewise, the professionals performing the DTIM assessments had had no clinical information about the patients and were not involved in the case conferences or informed about their results. These instruments and their applications have been described in detail by Rösner et al.^5^

### Statistical analysis

The statistical analysis was conducted using IBM SPSS Statistics version 21 and the *lavaan* package for R version 2022.12.0 build 353. There were no missing values.

The construct validity of the DTIM was assessed using a confirmatory factor analysis for ordinal data. The model for ordinal data was used because interpretation of the differences between scores (a key criterion for metric data) was not possible for the DTIM items or, consequently, for the domain scores. First, a one-factorial model with the seven domains as indicators for the overall results was assessed. In a next step, the seven domains ‘orientation’, ‘language’, ‘attention’, ‘memory’, ‘planning’, ‘abstract logical thinking’ and ‘visual perception’ were tested separately. The items of each domain were assumed to load on to a single factor, which was supposed to be the domain topic.

Model fit was evaluated using the chi-squared test, in which *P* > 0.05 was considered to indicate a ‘good model fit’. Furthermore, the root mean square error of approximation (RMSEA), standardised root mean square residual (SRMR) and comparative fit index (CFI) were calculated, as minor misfits, especially in large samples, may cause significant chi-squared values. The following cut-off scores were used to determine good model fit: RMSEA < 0.06 (excellent: <0.05; moderate: 0.08–0.1); SRMR < 0.08 (excellent: <0.05; moderate: 0.08–0.1); and CFI > 0.95 (excellent: >0.95; good: 0.9–0.95; moderate: 0.80–0.89).^24–26^

An item analysis was conducted. For item difficulty, mean item scores were divided by the item’s maximal possible score. Standard deviations were used as a measure of item variance, and discrimination values were calculated (part–whole corrected). Internal consistency was assessed using Cronbach’s α at the overall and domain levels. Values of Cronbach’s α above 0.7 are satisfactory, and those above 0.9 are excellent.^27,28^ Confidence intervals for Cronbach’s α were determined using the Feldt method. A retest assessment (T2) was conducted 6 months after the baseline assessment T1. Eighty-two patients without dementia participated in T2. As a measure of test–retest reliability, intraclass correlation (ICC) values were calculated in this sample. Test–retest reliability was further explored using Bland–Altman plots.

## Results

### Construct validity: confirmatory factor analysis at overall and domain levels

#### Overall level

A one-factor model with eight variables (domain sum scores) that loaded on to one factor (cognitive functioning) was tested in the confirmatory factor analysis. The one-factor model showed the following fit-indices: *χ*^2^ = 9.82, d.f. = 14, *P* = 0.775, RMSEA (90% CI) = 0.000 (0.000–0.065), CFI = 1.000 and SRMR = 0.026. The parameter estimates of the confirmatory factor analysis, including standardised and unstandardised estimates, standard errors, *z*-values, *P*-values and residual variances are summarised in Table 4. The factor loadings ranged from 0.81 (‘planning’) to 0.96 (‘language’).

**Table 4:** Parameter estimates of the confirmatory factor analysis (overall level)

DTIM domains	Domain scores, *M* (s.d.)	Unstandardised estimate	Standardised estimate (factor loadings)	s.e.	*z*	*P*-value	Residual variance
Orientation	4.83 (2.94)	1.00	0.90				0.19
Language	6.92 (4.1)	1.07	0.96	0.04	30.26	<0.001	0.08
Attention	5.92 (2.32)	0.96	0.86	0.03	29.45	<0.001	0.26
Memory	7.87 (5.05)	0.96	0.86	0.03	29.44	<0.001	0.26
Planning	14.89 (5.16)	0.90	0.81	0.03	28.01	<0.001	0.34
Abstract logical thinking	2.48 (1.86)	0.92	0.83	0.03	29.72	<0.001	0.32
Visual perception	4.15 (2.33)	0.94	0.84	0.03	28.92	<0.001	0.29

DTIM, Dementia Test for People with Intellectual Disability.

#### Domain level

The thresholds for the one-factor model for the items in the different domains that loaded on to the respective factors are given in the Supplementary Material available at https://doi.org/10.1192/bjo.2024.847.

##### Orientation

The estimation of a one-factor model with eight variables (items in the ‘orientation’ domain) loading on to one factor (‘orientation’) failed.

##### Language

A one-factor model with six variables (items in the ‘language’ domain) that loaded on to one factor (‘language’) was tested in the confirmatory factor analysis. The one-factor model fitted the data well: *χ*^2^ = 1.24, d.f. = 9, *P* = 0.999, RMSEA (90% CI) = 0.000 (0.000–0.000), CFI = 1.000 and SRMR = 0.099. The parameter estimates of the confirmatory factor analysis, including standardised and unstandardised estimates, standard errors, *z*-values, *P*-values and residual variances are summarised in Table 5. The factor loadings ranged from 0.72 to 0.93.

**Table 5:** Parameter estimates of the confirmatory factor analysis (‘language’ domain)

Items	Unstandardised estimate	Standardised estimate (factor loadings)	s.e.	*z*	*P*-value	Residual variance
2.1	1.00	0.79				0.38
2.2	0.92	0.72	0.11	8.63	<0.001	0.48
2.3	1.19	0.93	0.10	11.53	<0.001	0.13
2.4	1.15	0.90	0.09	12.63	<0.001	0.19
2.5	1.14	0.90	0.10	11.84	<0.001	0.19
2.6	1.17	0.92	0.10	11.42	<0.001	0.15

##### Attention

A one-factor model with five variables (items in the ‘attention’ domain) that loaded on to one factor (‘attention’) was tested in the confirmatory factor analysis. The model fit was mixed. The *χ*^2^ value was significant (*χ*^2^ = 20.996, d.f. = 5, *P* = 0.001), indicating some discrepancy between the model and the observed data. Other parameters (RMSEA (90% CI) = 0.174 (0.101–0.254), CFI = 0.935 and SRMR = 0.228) suggested fair to moderate fit. The parameter estimates of the confirmatory factor analysis, including standardised and unstandardised estimates, standard errors, *z*-values, *P*-values and residual variances are summarised in Table 6. The factor loadings ranged from 0.58 to 0.85.

**Table 6:** Parameter estimates of the confirmatory factor analysis (‘attention’ domain)

Items	Unstandardised estimate	Standardised estimate (factor loadings)	s.e.	*z*	*P*-value	Residual variance
3.1	1.00	0.85				0.29
3.2	0.68	0.58	0.17	4.04	<0.001	0.67
3.3	0.79	0.67	0.18	4.47	<0.001	0.55
3.4	0.97	0.82	0.18	5.44	<0.001	0.33
3.5	0.98	0.83	0.18	5.38	<0.001	0.32

##### Memory

A one-factor model with four variables (items in the ‘memory’ domain) that loaded on to one factor (‘memory’) was tested in the confirmatory factor analysis. The one-factor model fitted the data well: *χ*^2^ = 1.017, d.f. = 2, *P* = 0.601, RMSEA (90% CI) = 0.000 (0.000–0.158), CFI = 1.000 and SRMR = 0.028. The parameter estimates of the confirmatory factor analysis, including standardised and unstandardised estimates, standard errors, *z*-values, *P*-values and residual variances are summarised in Table 7. The factor loadings ranged from 0.74 to 0.95.

**Table 7:** Parameter estimates of the confirmatory factor analysis (‘memory’ domain)

Items	Unstandardised estimate	Standardised estimate (factor loadings)	s.e.	*z*	*P*-value	Residual variance
4.1	1.00	0.91				0.18
4.2	1.05	0.95	0.08	12.68	<0.001	0.09
4.3	0.97	0.88	0.06	15.84	<0.001	0.22
4.4	0.82	0.74	0.08	10.60	<0.001	0.45

##### Planning

A one-factor model with nine variables (items in the ‘planning’ domain) that loaded on to one factor (‘planning’) was tested in the confirmatory factor analysis. The one-factor model fitted the data well, although the SRMR parameter was high compared with those of ideal fits: *χ*^2^ = 25.037, d.f. = 27, *P* = 0.572, RMSEA (90% CI) = 0.000 (0.000–0.069), CFI = 1.000 and SRMR = 0.145. The parameter estimates of the confirmatory factor analysis, including standardised and unstandardised estimates, standard errors, *z*-values, *P*-values and residual variances, are summarised in Table 8. The factor loadings ranged from 0.61 to 0.87.

**Table 8:** Parameter estimates of the confirmatory factor analysis (‘planning’ domain)

Items	Unstandardised estimate	Standardised estimate (factor loadings)	s.e.	*z*	*P*-value	Residual variance
5.1	1.00	0.69				0.53
5.2	0.89	0.61	0.16	5.66	<0.001	0.63
5.3	1.01	0.69	0.13	7.61	<0.001	0.52
5.4	1.25	0.86	0.14	8.95	<0.001	0.27
5.5	1.21	0.83	0.14	8.92	<0.001	0.31
5.6	1.25	0.86	0.14	8.87	<0.001	0.26
5.7	1.18	0.81	0.13	8.87	<0.001	0.34
5.8	1.25	0.86	0.15	8.31	<0.001	0.26
5.9	1.27	0.87	0.16	8.12	<0.001	0.24

##### Abstract logical thinking

A one-factor model with five variables (items in the ‘abstract logical thinking’) that loaded on to one factor (‘abstract logical thinking’) was tested in the confirmatory factor analysis. The one-factor model fitted the data well: χ^2^ = 2.979, d.f. = 5, *P* = 0.703, RMSEA (90% CI) = 0.000 (0.000–0.102), CFI = 1.000 and SRMR = 0.063. The parameter estimates of the confirmatory factor analysis, including standardised and unstandardised estimates, standard errors, *z*-values, *P*-values and residual variances are summarised in Table 9. The factor loadings ranged from 0.61 to 0.83.

**Table 9:** Parameter estimates of the confirmatory factor analysis (‘abstract logical thinking’ domain)

Items	Unstandardised estimate	Standardised estimate (factor loadings)	s.e.	*z*	*P*-value	Residual variance
6.1	1.00	0.61				0.62
6.2	1.08	0.66	0.24	4.56	<0.001	0.56
6.3	1.34	0.82	0.28	4.72	<0.001	0.33
6.4	1.30	0.79	0.27	4.87	<0.001	0.37
6.5	1.35	0.83	0.28	4.86	<0.001	0.31

##### Visual perception

A one-factor model with six variables (items in the ‘visual perception’ domain) that loaded on to one factor (‘visual perception’) was tested in the confirmatory factor analysis. The one-factor model showed a reasonably good fit: *χ*^2^ = 25.337, d.f. = 9, *P* = 0.003, RMSEA (90% CI) = 0.131 (0.072–0.192), CFI = 0.995 and SRMR = 0.114. The parameter estimates of the confirmatory factor analysis, including standardised and unstandardised estimates, standard errors, *z*-values, *P*-values and residual variances are summarised in Table 10. The factor loadings ranged from 0.66 to 1.02.

**Table 10:** Parameter estimates of the confirmatory factor analysis (‘visual perception’ domain)

Items	Unstandardised estimate	Standardised estimate (factor loadings)	s.e.	*z*	*P*-value	Residual variance
7.1	1.00	0.91				0.17
7.2	1.12	10.02	0.07	15.44	<0.001	−0.04
7.3	1.04	0.94	0.05	21.96	<0.001	0.12
7.4	0.84	0.76	0.07	12.11	<0.001	0.42
7.5	0.73	0.66	0.07	9.88	<0.001	0.57
7.6	0.94	0.86	0.07	13.24	<0.001	0.27

### Item analysis: item difficulty, variance and discrimination

In the ‘orientation’ domain, item difficulties ranged from 0.31 to 0.87 in the control group and from 0.19 to 0.75 in the dementia group. Overall, a wide range of difficulties was covered, and ceiling and floor effects were avoided. The items’ standard deviations ranged from 0.46 to 0.60 in the control group and from 0.40 to 0.82 in the dementia group. The discrimination values were moderate, ranging from 0.50 to 0.74 in the control group and from 0.23 to 0.65 in the dementia group.

In the ‘language’ domain, item difficulties ranged from 0.26 to 0.86 in the control group and from 0.19 to 0.81 in the dementia group. Overall, a wide range of difficulties was covered, and ceiling and floor effects were avoided. The items’ standard deviations ranged from 0.35 to 1.42 in the control group and from 0.40 to 1.40 in the dementia group. The discrimination values were moderate, ranging from 0.56 to 0.76 in the control group and from 0.48 to 0.73 in the dementia group.

In the ‘attention’ domain, item difficulties ranged from 0.45 to 0.98 in the control group and from 0.31 to 0.94 in the dementia group. Overall, a wide range of difficulties was covered, and ceiling and floor effects were avoided, although one item (5.1) was solved by almost all participants in both groups. The items’ standard deviations were weak to moderate and ranged from 0.15 to 1.24 in the control group and from 0.25 to 1.32 in the dementia group. The discrimination values ranged from 0.07 to 0.60 in the control group and from 0.34 to 0.72 in the dementia group.

In the ‘memory’ domain, item difficulties ranged from 0.60 to 0.70 in the control group and from 0.10 to 0.44 in the dementia group. The items seemed to be more difficult in the dementia group, and floor as well as ceiling effects could be avoided. The items’ standard deviations ranged from 0.49 to 2.54 in the control group and from 0.51 to 1.63 in the dementia group. The discrimination values were moderate, ranging from 0.53 to 0.78 in the control group and from 0.68 to 0.73 in the dementia group.

In the ‘planning’ domain, item difficulties ranged from 0.62 to 0.99 in the control group and from 0.37 to 1.00 in the dementia group. Overall, the items’ difficulties were moderate to high, suggesting that the participants in both groups were mostly able to solve the tasks. The items’ standard deviations ranged from 0.1 to 2.17 in the control group and from 0.00 to 2.48 in the dementia group. The discrimination values were mostly weak to moderate, ranging from 0.28 to 0.73 in the control group and from 0.02 to 0.86 in the dementia group. No discrimination value could be calculated for item 5.2 in the dementia group owing to zero variance.

In the ‘abstract logical thinking’ domain, item difficulties ranged from 0.14 to 0.69 in the control group and from 0.06 to 0.50 in the dementia group. The difficulties were low to moderate, indicating that some items were challenging for the participants in both groups. The items’ standard deviations ranged from 0.35 to 0.89 in the control group and from 0.25 to 0.98 in the dementia group. The discrimination values were weak to moderate, ranging from 0.39 to 0.51 in the control group and from 0.23 to 0.59 in the dementia group.

In the ‘visual perception’ domain, item difficulties ranged from 0.38 to 0.79 in the control group and from 0.28 to 0.69 in the dementia group. Overall, a wide range of difficulties was covered, and ceiling and floor effects were avoided. The items’ standard deviations ranged from 0.41 to 0.83 in the control group and from 0.48 to 0.73 in the dementia group. The discrimination values were fair to moderate, ranging from 0.40 to 0.75 in the control group and from 0.36 to 0.78 in the dementia group.

Item difficulties, standard deviations and discriminations for each item are summarised in Table 3.

### Reliability analysis

#### Internal consistency: Cronbach’s α at overall and domain levels

The results of the internal consistency analysis are summarised in Table 11. Overall, Cronbach’s α ranged from 0.94 (dementia) to 0.95 (no dementia). At the domain level, it varied between 0.53 (abstract logical thinking, dementia group) and 0.87 (orientation, no-dementia group). Details including 95% confidence intervals are displayed in Table 11.

**Table 11:** Internal consistency at overall and domain levels

Domain	Cronbach’s α (95% CI)	
	Control group	Dementia
Orientation	0.87 (0.83–0.91)	0.79 (0.59–0.91)
Language	0.79 (0.72–0.85)	0.79 (0.58–0.92)
Attention	0.57 (0.41–0.69)	0.66 (0.31–0.87)
Memory	0.76 (0.67–0.83)	0.81 (0.60–0.93)
Planning	0.77 (0.69–0.83)	0.74 (0.49–0.89)
Abstract logical thinking	0.67 (0.55–0.77)	0.53 (0.02–0.81)
Visual perception	0.81 (0.74–0.86)	0.84 (0.69–0.94)
Overall	0.95 (0.93–0.96)	0.94 (0.89–0.98)

#### Test–retest reliability (T1 versus T2, control group)

**Fig. 2:**
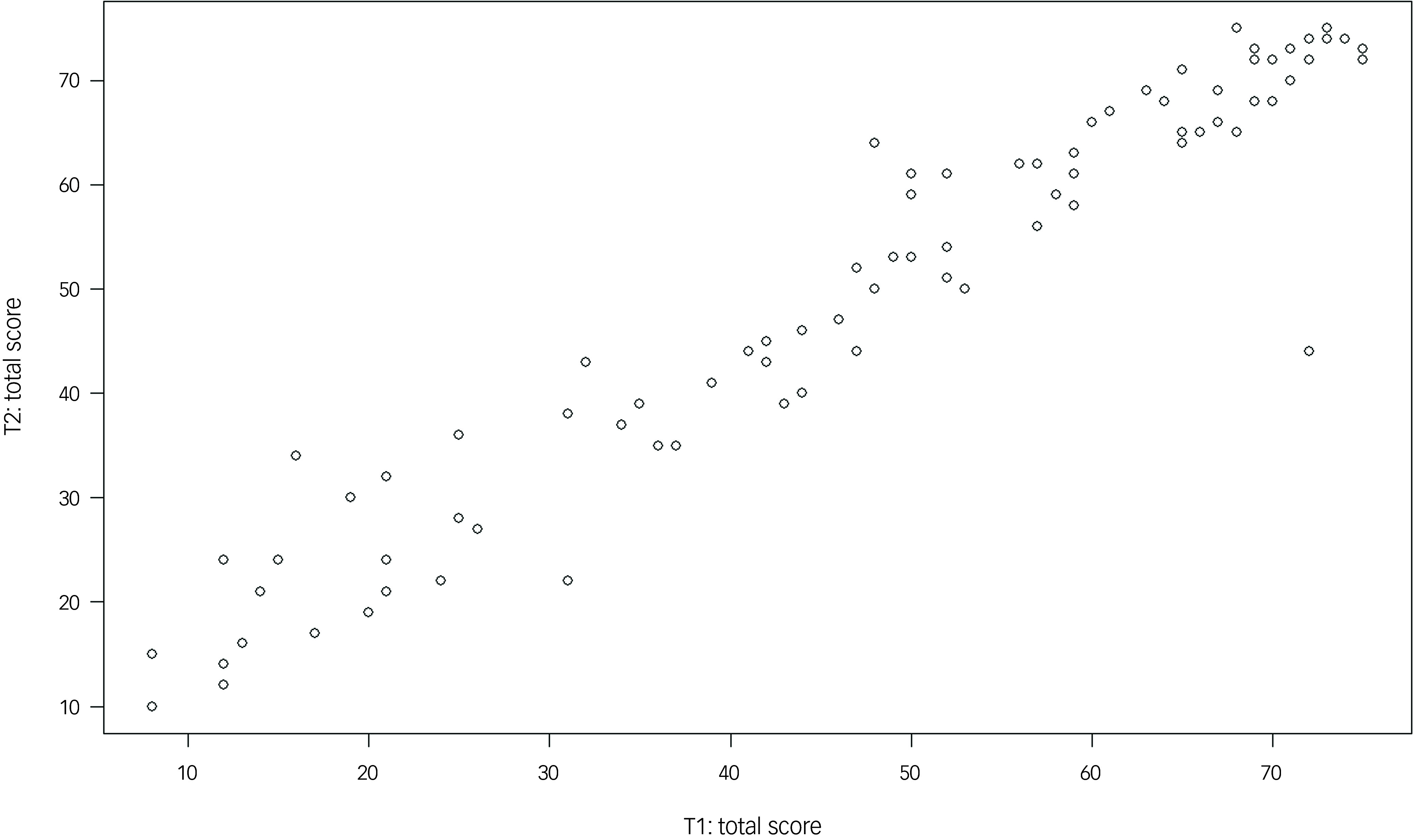
Test–retest reliability for the Dementia Test for People with Intellectual Disability total score.

In total, 95 of 107 participants completed the second assessment (T2) 6 months after T1. On average, participants without dementia (*n* = 82) scored 2.44 points higher at T2 compared with T1 (s.d. = 5.77). Participants in the dementia group (*n* = 13) scored on average 0.31 points lower at T2 compared with T1 (s.d. = 3.99). As a decline in test scores was expected in the dementia group, test–retest reliability was calculated only for the 82 participants without dementia who were tested at T1 and T2. The ICC was 0.95 for the total score (*P* < 0.001, 95% CI: 0.93–0.95). Figure 2 provides a graphical representation of the test–retest reliability for the total score. At the domain level, the ICC values for the sum scores of the domains were highest for ‘language’ (ICC = 0.96, *P* < 0.001, 95% CI: 0.94–0.97), ‘orientation’ (ICC = 0.92; *P* < 0.001, 95% CI: 0.88–0.95) and ‘planning’ (ICC = 0.90, *P* < 0.001, 95% CI: 0.81–0.94); still good for ‘memory’ (ICC = 0.85; *P* < 0.001, 95% CI: 0.77–0.90), ‘attention’ (ICC = 0.84; *P* < 0.001, 95% CI: 0.76–0.89) and ‘visual perception’ (ICC = 0.82; *P* < 0.001, 95% CI: 0.73–0.88); and lowest for ‘abstract logical thinking’ (ICC = 0.69; *P* < 0.001, 95% CI: 0.55–0.79).

**Fig. 3:**
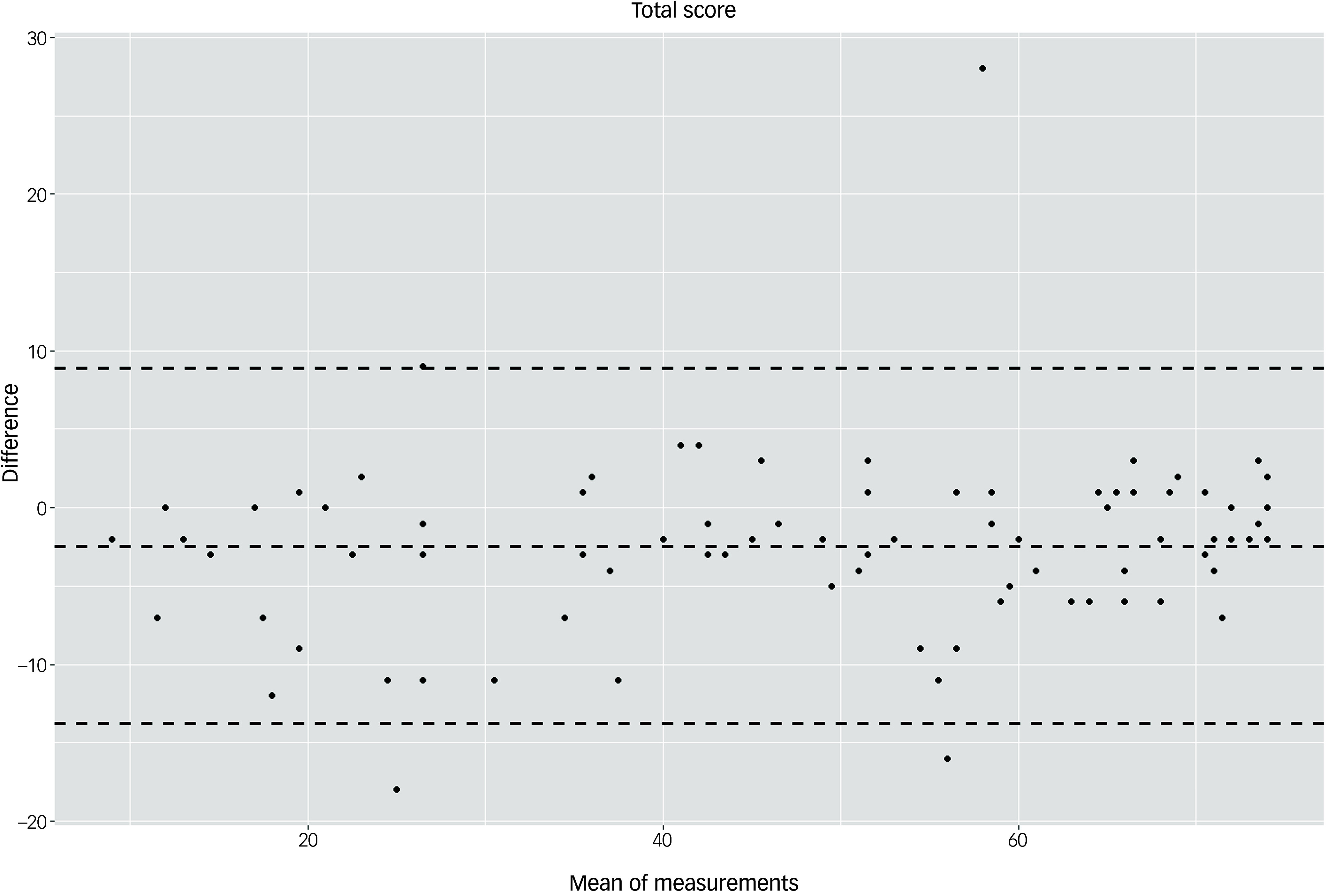
Bland–Altman plot for the Dementia Test for People with Intellectual Disability total score.

**Fig. 4:**
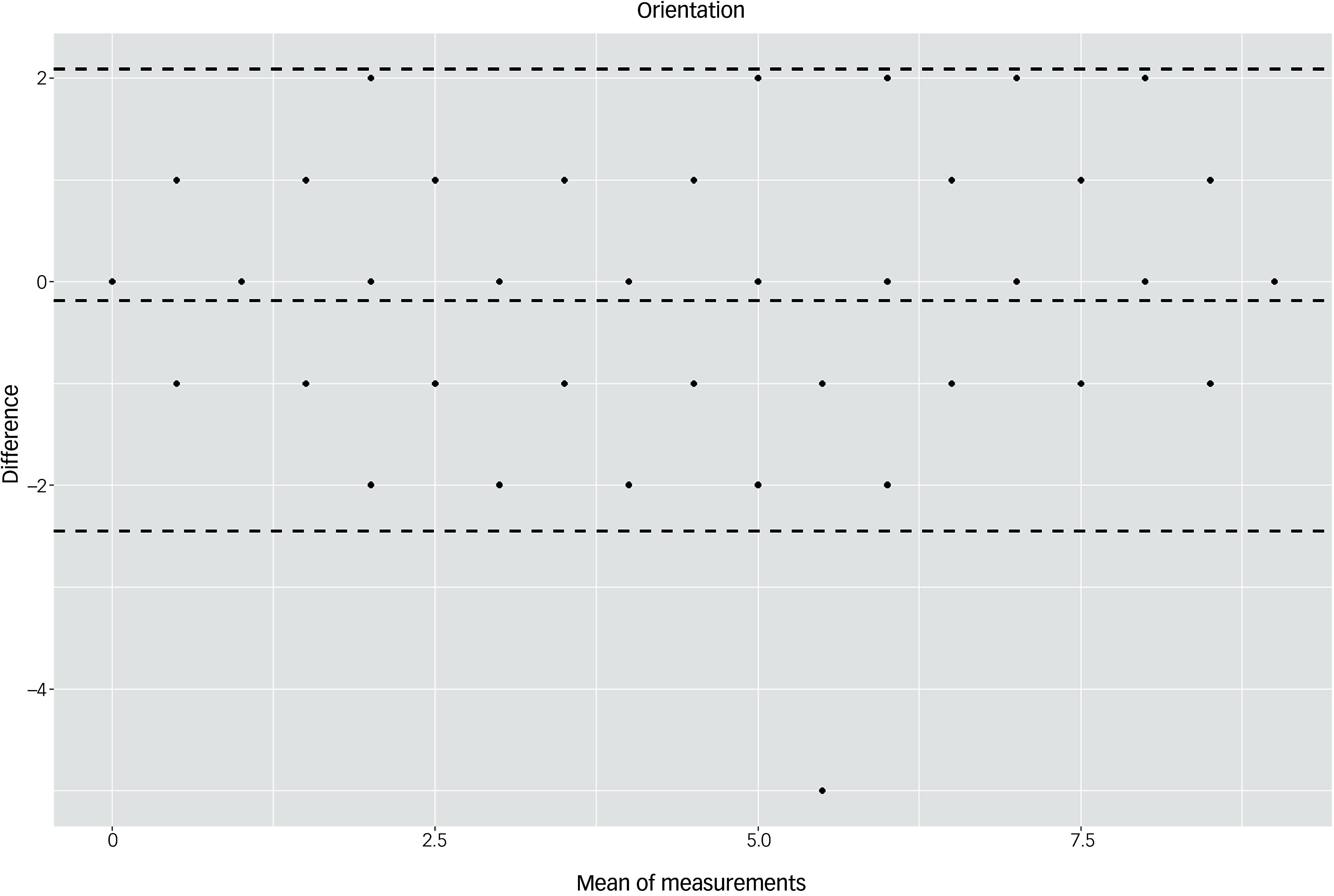
Bland–Altman plot for ‘orientation’ domain.

**Fig. 5:**
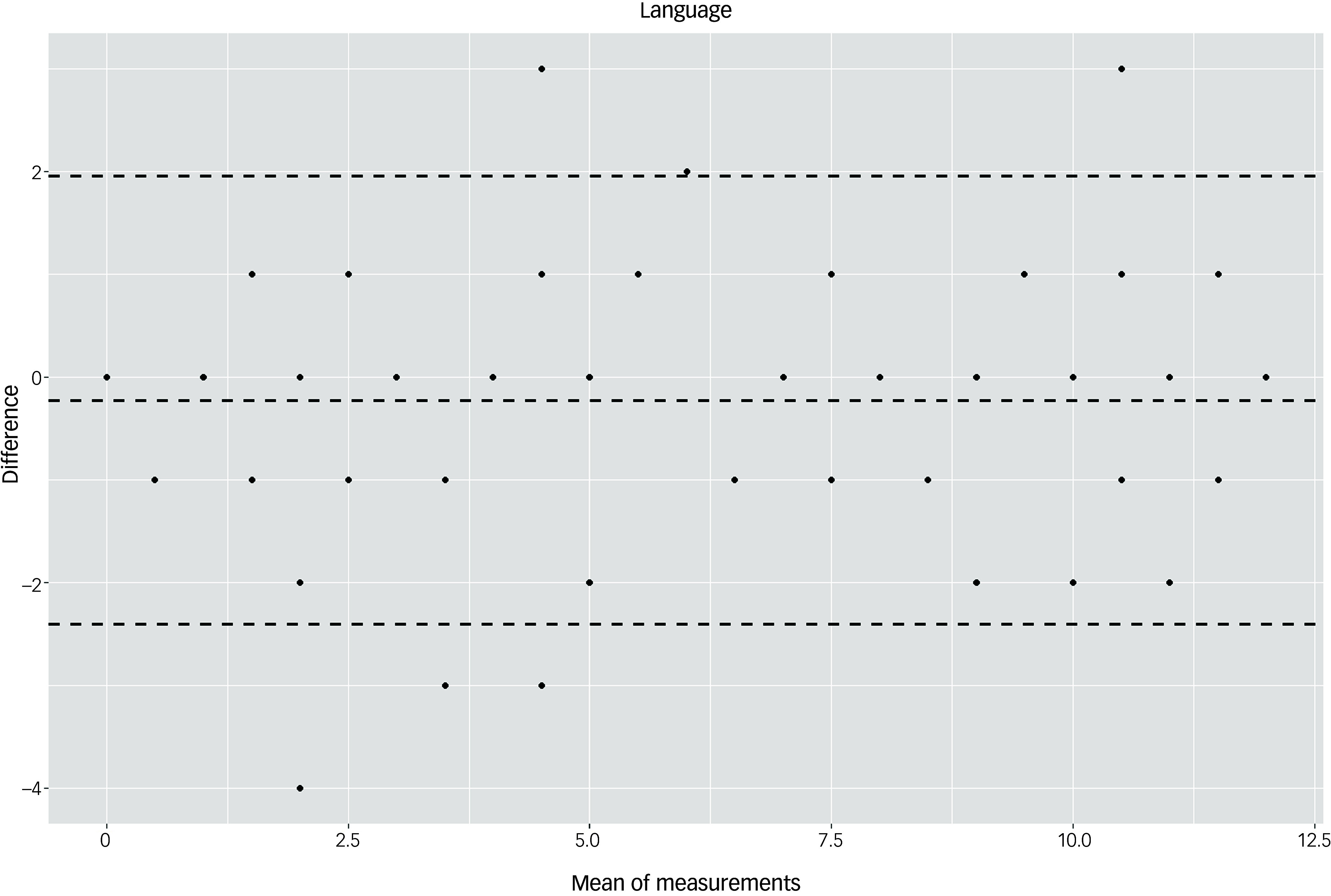
Bland–Altman plot for ‘language’ domain.

**Fig. 6:**
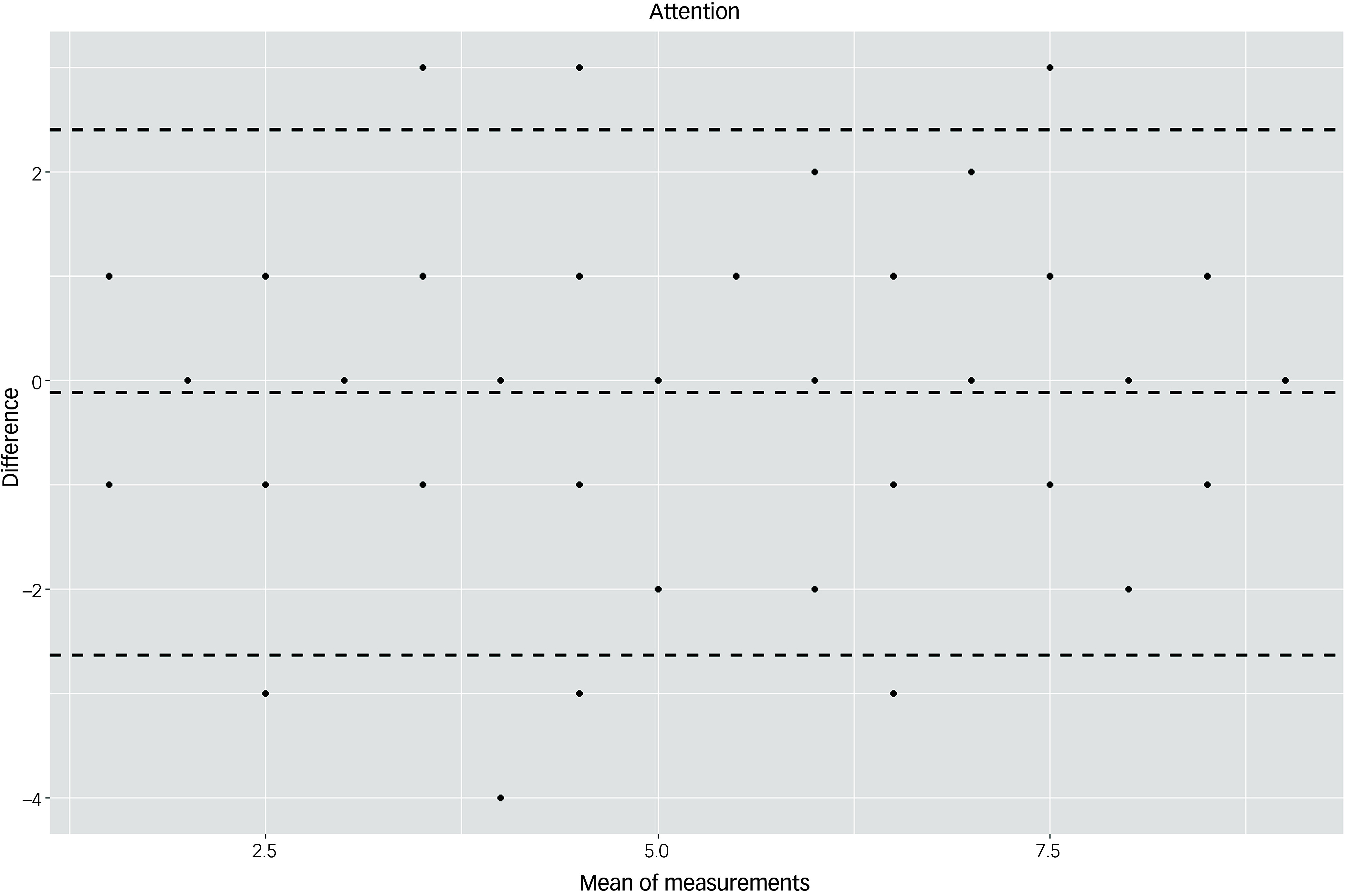
Bland–Altman plot for ‘attention’ domain.

**Fig. 7:**
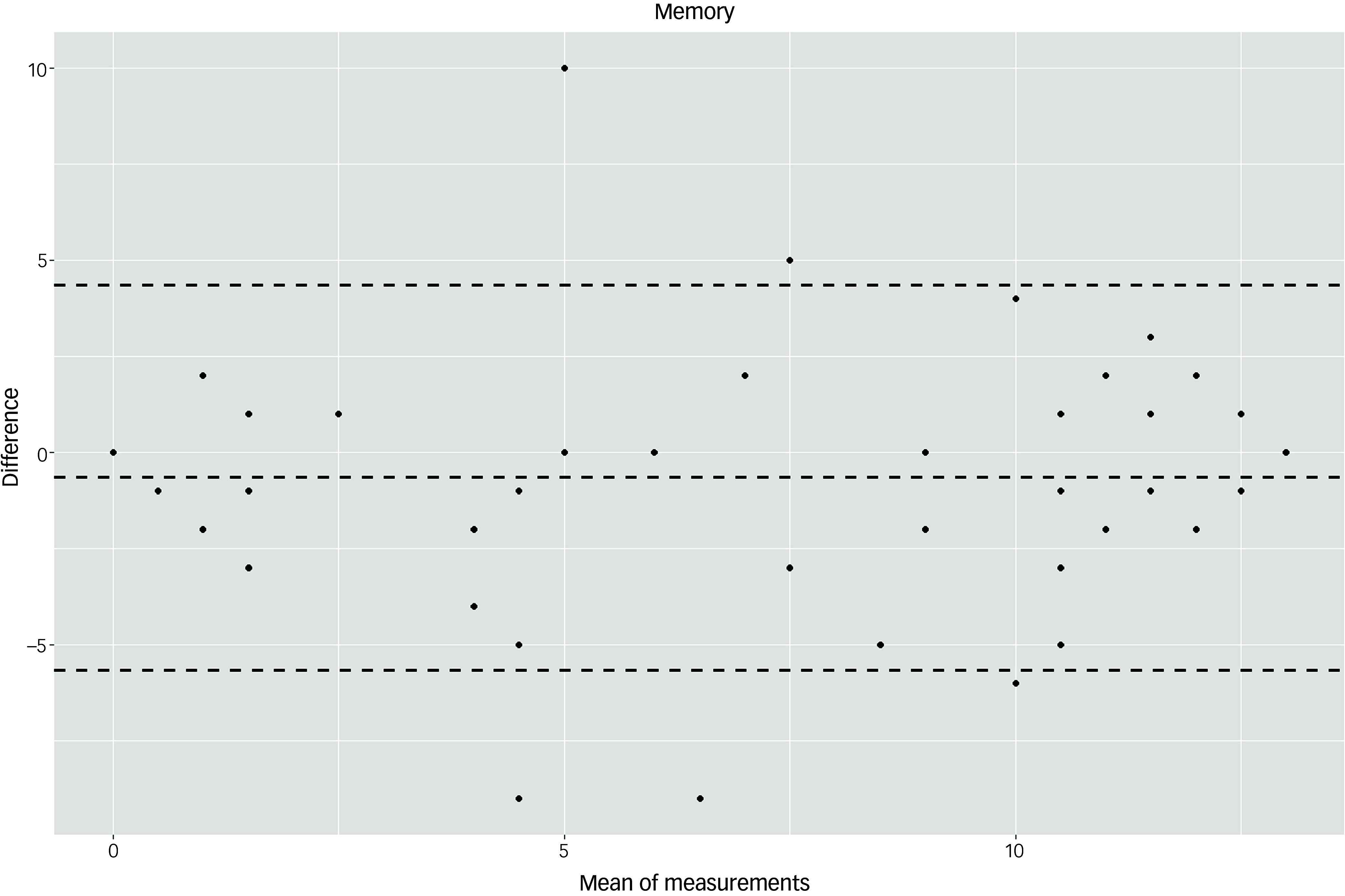
Bland–Altman plot for ‘memory’ domain.

**Fig. 8:**
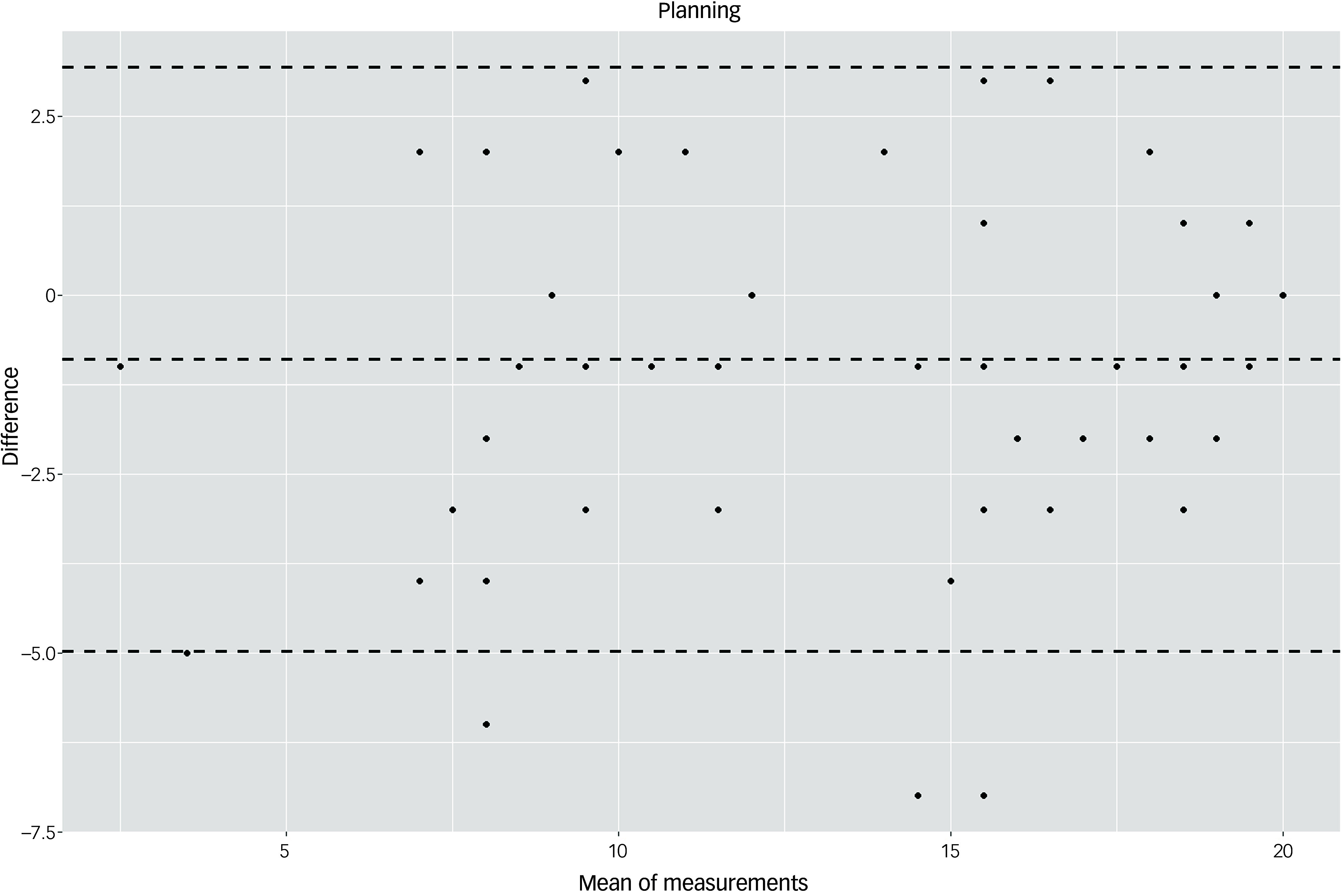
Bland–Altman plot for ‘planning’ domain.

**Fig. 9:**
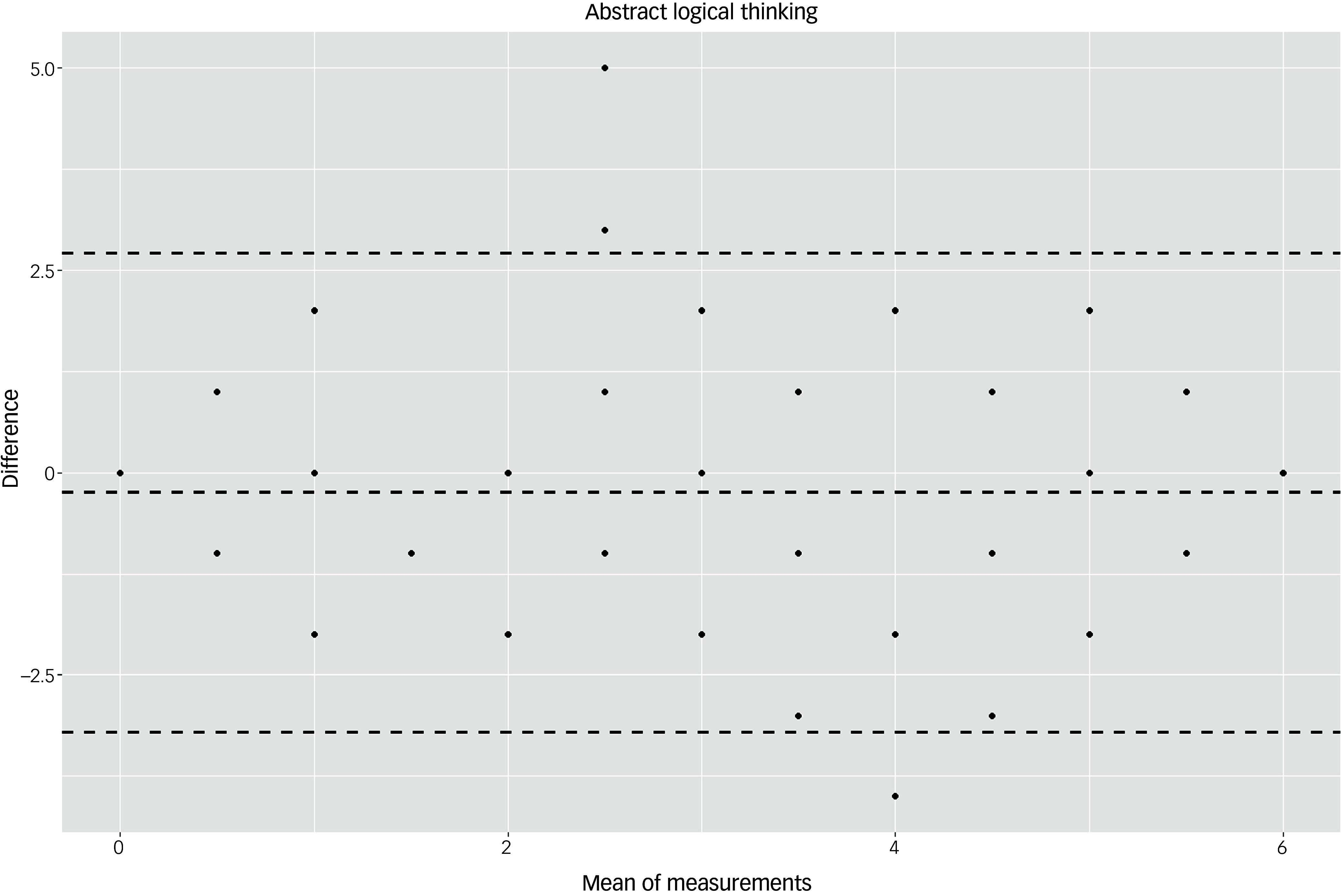
Bland–Altman plot for ‘abstract logical thinking’ domain.

**Fig. 10:**
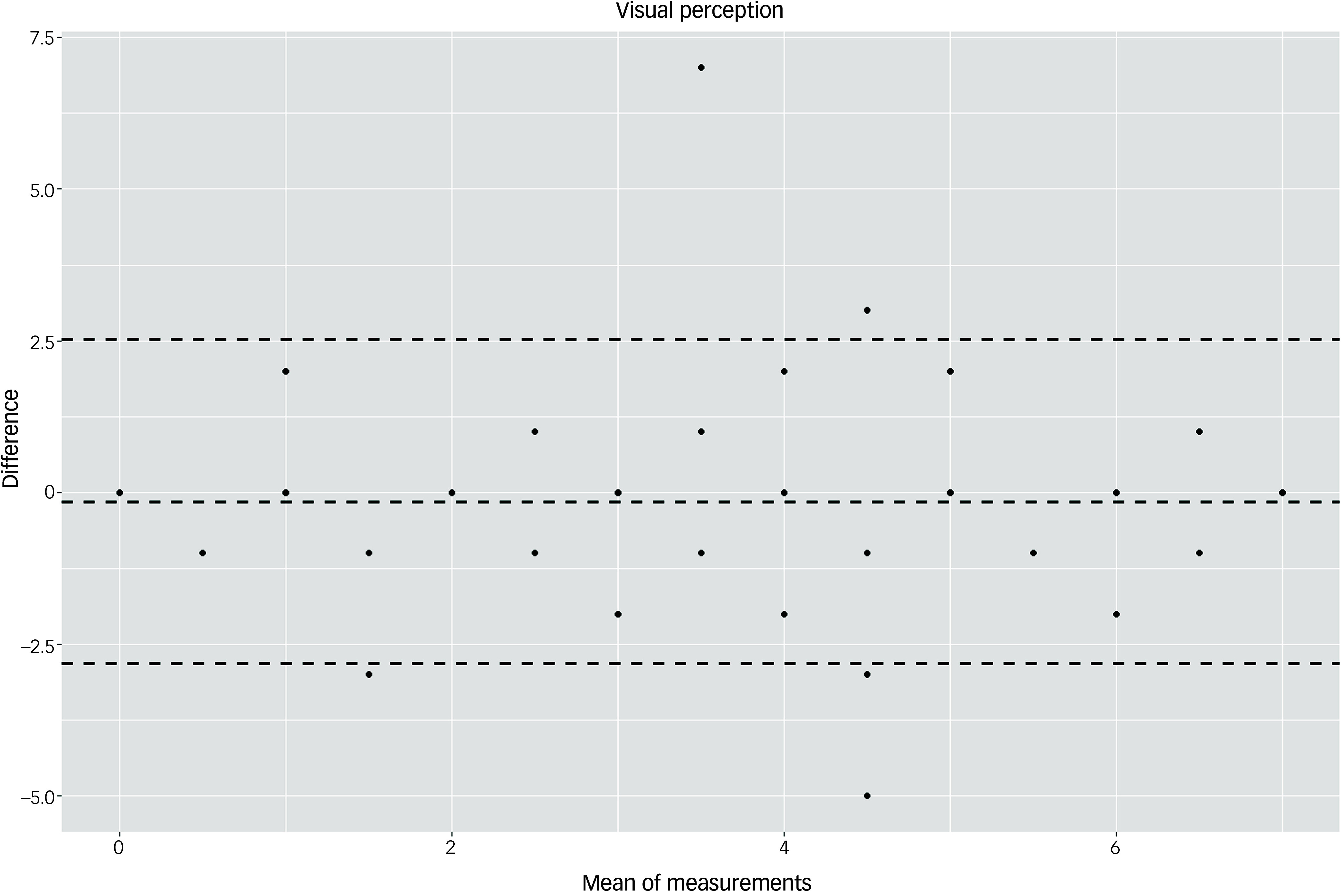
Bland–Altman plot for ‘visual perception’ domain.

The Bland–Altman plots at the domain level and for the total score supported the results of the test–retest reliability analysis (Figs. 3–10). For the total score and for all domain scores, the bias was close to 0. The majority of data points clustered within the 95% limits of agreement; however, several outliers were observed. These findings suggest good to moderate test–retest reliability for the total score as well as at the domain level.

## Discussion

Various assessment instruments have been developed for diagnosis of dementia in people with intellectual disability; however, diagnostic validity has not yet been assessed systematically for most of these measures.^6,29^ Experts suggest the use of complementary test batteries that cover both the direct assessment of cognitive abilities and third-party reports of daily functioning.^29^ Moreover, sequential assessments are recommended to ascertain any cognitive decline.^6^

The DTIM is a newly developed neuropsychological test battery for assessment of the current state of cognitive functioning in people with an intellectual disability and suspected dementia.^19^ It should be complemented by an informant-based interview (DSQIID).^19^ As no norm data are available for any of the neuropsychological test batteries designed for people with intellectual disabilities, repeated assessments are necessary to observe potential changes in cognitive functions over time. Thus, a decline in DTIM scores in subsequent assessments objectifies the cognitive decline and supports the suspicion of dementia. Improved or stable DTIM scores indicate no cognitive decline and reject the suspicion of dementia. The first assessment measures the initial or premorbid level of cognitive functioning, thereby creating a reference for subsequent assessments. The results of the initial assessment represent the variability of cognitive abilities in the target population of the DTIM: higher scores are expected in individuals with milder forms of intellectual disability.

The construct ‘cognitive functioning’ as assessed by DTIM includes cognitive processes that are typically affected in patients with suspected dementia.^20^ Specifically, the DTIM tasks are grouped in the following domains: orientation, language, attention, memory, planning, abstract logical thinking and visual perception.

The confirmatory factor analysis of the neuropsychological test battery showed a one-factor model which fitted the data well. At the domain level, a one-factor model showed a good fit index for the four domains ‘language’, ‘memory’, ‘planning’ and ‘abstract logical thinking’; a reasonable fit index for ‘visual perception’; and a mixed model-fit for ‘attention’; and it failed for ‘orientation’. Internal consistency as assessed with Cronbach’s α indicated excellent reliability at the overall scale level. As intended, item analysis revealed a wide range of difficulties, and ceiling and floor effects were avoided. The test–retest reliability for the group without dementia between T1 and T2 was excellent, with an ICC of 0.95 for the total score. At the domain level, the ICC values for the sum scores ranged from 0.69 for ‘abstract logical thinking’ to 0.96 for ‘language’. The low test–retest reliability for ‘abstract logical thinking’ could have been due to the type of task. Here, we used so-called novelty tasks, in which the generation of solutions is required and repetition does not make sense.

### Construct validity

The one-factor model was supported by a confirmatory factor analysis which fitted the data well. However, at the domain level, confirmation failed in the ‘orientation’ domain, and only a mixed model-fit could be found in the ‘attention’ domain. This may have been owing to the small sample size. Furthermore, some tasks may require more than one cognitive process, leading to a poorer model fit. For example, repeating increasingly long sequences of numbers (item 3.5 in the ‘attention’ domain) requires the ability to speak, knowledge of numbers, and verbal memory span, in addition to attention. Nevertheless, the confirmatory factor analysis at the overall level had a good model fit, suggesting that DTIM items adequately represent one underlying latent factor, which we believe to be cognitive functioning. Very few groups have examined the construct validity of their scales. Deb et al^11^ found a four-factor model of the DSQIID in an exploratory factor analysis. Startin et al^17^ described a five-factor model for the CS-DS. Both instruments are informant-based and involve no direct measures of cognitive functions.

### Item analysis

Item difficulties ranged from 0.14 to 0.98; most items scored in the middle range, as is preferable according to Bortz and Döring.^30^ Item difficulty has rarely been evaluated in former validity analyses of direct assessment instruments for suspected dementia in people with an intellectual disability. The abilities of the items to discriminate between those with and those without dementia were moderate in the ‘orientation’, ‘language’, ‘attention’ and ‘memory’ domains; fair to moderate in the ‘visual perception’ domain; and weak to moderate in the ‘planning’ and ‘abstract logical thinking’ domains. Effects on the visual perception, planning and abstract logical reasoning cognitive functions are not among the prominent and early symptoms of dementia in people with intellectual disability, so one would not expect high discriminatory power here.

### Internal consistency

The internal consistency indicated excellent reliability at the overall scale level for the dementia group (Cronbach’s α = 0.94) and for the group without dementia (Cronbach’s α = 0.95). These values are comparable with the internal consistencies of other dementia assessment instruments, including the CS-DS (Cronbach’s α = 0.96)^17^ or the Prudhoe Cognitive Function Test (Cronbach’s α = 0.96).^31^ However, at the domain level, weaknesses were observed for the ‘abstract logical thinking’ domain (Cronbach’s α: 0.53–0.67) and ‘attention’ domain (Cronbach’s α: 0.57–0.66); the other domains consistently had values >0.74, indicating sufficient homogeneity of the scale at the domain level. Lower values at the domain level may result from the complexity and heterogeneity of the tested constructs, as well as from a small number of items within one domain.

### Test–retest reliability

According to the descriptive statistical analysis, participants in the control group scored on average 2.44 points higher at T2 compared with T1. This increase in test scores could possibly be explained by learning effects. In the dementia group, the results remained stable: there was an average decrease of 0.33 points, considerably less than the cut-off of 5 points indicating cognitive decline according to Kuske and Müller.^22^ These results suggest that an interval of 6 months is not enough to capture the cognitive decline due to dementia. However, as we expected scores to change over time in the dementia group owing to the progredient nature of the disease, we calculated test–retest reliability only for the control group. The ICC values were 0.95 for the total score and >0.82 in six of seven domains. Likewise, the Bland–Altman plots suggested good test–retest reliability: the bias was close to 0 for the total score as well as for domain scores, and most values clustered within the 95% limits of agreement. Overall, the values indicated good to excellent test–retest reliability. Reliability has been analysed for few tools,^6^ especially direct assessment instruments such as the Cambridge Examination for Mental Disorders of the Elderly, modified for use assessing people with Down Syndrome; the Cambridge Cognitive Examination, modified for use in a group with Down syndrome; the Down Syndrome Mental Status Examination; the Mini-Mental State Examination; and the NeuroTrax Computerized Moderate to Severe Impairment Battery. The Test for Severe Impairment has some evidence of reliability; however, this was obtained using a small sample size. The Severe Impairment Battery has shown a high test–retest reliability in a group of people with intellectual disability without dementia, although this research was conducted several years ago.^32^ For the Prudhoe Cognitive Function Test,^31^ very high ICC values (0.99–0.98) have been reported.^33^ The test–retest reliability of the DTIM was comparable with that of the newly developed CS-DS,^17^ for which an ICC value of 0.95 at the scale level was reported in a study of 36 individuals with Down syndrome.

### Limitations

Some participants dropped out between T1 and T2 owing to follow-up consultations being missed. The difficulties in follow-up were mostly caused by logistic factors, e.g. the coronavirus pandemic, difficulties with transport or shortage of accompanying persons. Moreover, there was a large difference between the sample sizes for people with intellectual disability and dementia and those with no dementia, with the number of people with both intellectual disability and dementia being smaller.

At the domain level, the internal consistency was satisfactory to excellent in both samples (with and without dementia), except for the ‘abstract logical thinking’ and ‘attention’ domains. Weaknesses could also be seen in the confirmatory factor analysis in the ‘orientation’ and, again, ‘attention’ domains. Therefore, adaptation of these domains, especially the ‘attention’ domain, is recommended in any revision of the scale. A few items showed weak to fair discriminative power. These could be rephrased to improve the diagnostic value of the scale. Owing to the small sample size, the invariance across key demographic characteristics such as gender, migration status and severity of intellectual disability could not be evaluated.

In addition to cognitive functioning, several factors may influence a participant’s ability to perform a task (e.g. sensory or motor issues). This may raise concerns about the appropriateness of the confirmatory factor analysis for our analysis. To minimise the influence of such factors on the test results, participants with severe sensory impairments were excluded from the study.

A strength of the study was the blinding of the psychologist who applied the DTIM to the independent results of the case conference for or against dementia. The prospective design was a further strength of the study.

In their systematic review of measures for assessment of dementia in people with an intellectual disability, Zeilinger et al^16^ considered several instruments which were not developed for this target population or designed specifically for the assessment of dementia (e.g. intelligence tests). These instruments were neither specific enough for people with an intellectual disability nor for the disorder itself. Therefore, the authors recommended the application of instruments that were specifically developed or adapted for this population, which is the case for the DTIM. The results of the present study support its usage and proves its construct and item validity, internal consistency and test–retest reliability.

In the present study, we investigated people with intellectual impairment with dementia. People with an intellectual disability are a very heterogeneous group with different cognitive impairments. Dementia, a progressive disease with three stages, also shows very heterogeneous patterns of impairment. This fundamentally complicates dementia diagnostics and, accordingly, test development for this group of people.

## Supporting information

Sappok et al. supplementary material Sappok et al. supplementary material

## Data Availability

The data that support the findings of this study are available from the corresponding author, T.S., upon reasonable request.
