# Supplementary material for: Construct validity and reliability of the Dementia Test for People with Intellectual Disability: neuropsychological test battery for assessing cognitive functioning in people with intellectual disability
Source: BJPsych Open. 2025 Mar 24;11(2):e69. doi: 10.1192/bjo.2024.847 (PMC12001923; doi:10.1192/bjo.2024.847)
Supplement: Sappok et al. supplementary material [file S2056472424008470sup001.docx]

Supplement

Overall level: Thresholds for the one-factor model with eight variables (domain sum scores) that load onto one factor (cognitive functioning)

| Domain | Estimate | Standard error | z-value | *p* |
| --- | --- | --- | --- | --- |
| Orientation total score \|1 | -1.441 | 0.181 | -7.967 | 0.000 |
| Orientation total score \|2 | -1.079 | 0.151 | -7.132 | 0.000 |
| Orientation total score \|3 | -0.610 | 0.130 | -4.678 | 0.000 |
| Orientation total score \|4 | -0.297 | 0.124 | -2.402 | 0.016 |
| Orientation total score \|5 | -0.012 | 0.122 | -0.096 | 0.923 |
| Orientation total score \|6 | 0.200 | 0.123 | 1.635 | 0.102 |
| Orientation total score \|7 | 0.397 | 0.125 | 3.167 | 0.002 |
| Orientation total score \|8 | 0.638 | 0.131 | 4.865 | 0.000 |
| Orientation total score \|9 | 0.961 | 0.145 | 6.650 | 0.000 |
| Language total score \|1 | -1.320 | 0.169 | -7.795 | 0.000 |
| Language total score \|2 | -1.122 | 0.154 | -7.282 | 0.000 |
| Language total score \|3 | -0.855 | 0.139 | -6.135 | 0.000 |
| Language total score \|4 | -0.610 | 0.130 | -4.678 | 0.000 |
| Language total score \|5 | -0.422 | 0.126 | -3.357 | 0.001 |
| Language total score \|6 | -0.322 | 0.124 | -2.593 | 0.010 |
| Language total score \|7 | -0.200 | 0.123 | -1.635 | 0.102 |
| Language total score \|8 | 0.012 | 0.122 | 0.096 | 0.923 |
| Language total score \|9 | 0.224 | 0.123 | 1.827 | 0.068 |
| Language total score \|10 | 0.297 | 0.124 | 2.402 | 0.016 |
| Language total score \|11 | 0.610 | 0.130 | 4.678 | 0.000 |
| Language total score \|12 | 0.925 | 0.143 | 6.481 | 0.000 |
| Attention total score \|1 | -2.352 | 0.372 | -6.321 | 0.000 |
| Attention total score \|2 | -1.677 | 0.210 | -7.995 | 0.000 |
| Attention total score \|3 | -1.168 | 0.157 | -7.425 | 0.000 |
| Attention total score \|4 | -0.855 | 0.139 | -6.135 | 0.000 |
| Attention total score \|5 | -0.638 | 0.131 | -4.865 | 0.000 |
| Attention total score \|6 | -0.346 | 0.124 | -2.785 | 0.005 |
| Attention total score \|7 | -0.059 | 0.122 | -0.481 | 0.630 |
| Attention total score \|8 | 0.474 | 0.127 | 3.737 | 0.000 |
| Attention total score \|9 | 1.441 | 0.181 | 7.967 | 0.000 |
| Memory total score \|1 | -0.961 | 0.145 | -6.650 | 0.000 |
| Memory total score \|2 | -0.855 | 0.139 | -6.135 | 0.000 |
| Memory total score \|3 | -0.667 | 0.132 | -5.050 | 0.000 |
| Memory total score \|4 | -0.582 | 0.130 | -4.491 | 0.000 |
| Memory total score \|5 | -0.500 | 0.127 | -3.926 | 0.000 |
| Memory total score \|6 | -0.422 | 0.126 | -3.357 | 0.001 |
| Memory total score \|7 | -0.297 | 0.124 | -2.402 | 0.016 |
| Memory total score \|8 | -0.273 | 0.123 | -2.210 | 0.027 |
| Memory total score \|9 | -0.153 | 0.122 | -1.250 | 0.211 |
| Memory total score \|10 | -0.059 | 0.122 | -0.481 | 0.630 |
| Memory total score \|11 | 0.153 | 0.122 | 1.250 | 0.211 |
| Memory total score \|12 | 0.346 | 0.124 | 2.785 | 0.005 |
| Memory total score \|13 | 0.527 | 0.128 | 4.115 | 0.000 |
| Planning total score \|1 | -2.352 | 0.372 | -6.321 | 0.000 |
| Planning total score \|2 | -1.910 | 0.249 | -7.664 | 0.000 |
| Planning total score \|3 | -1.511 | 0.188 | -8.016 | 0.000 |
| Planning total score \|4 | -1.320 | 0.169 | -7.795 | 0.000 |
| Planning total score \|5 | -1.168 | 0.157 | -7.425 | 0.000 |
| Planning total score \|6 | -1.038 | 0.149 | -6.976 | 0.000 |
| Planning total score \|7 | -0.889 | 0.141 | -6.309 | 0.000 |
| Planning total score \|8 | -0.789 | 0.137 | -5.780 | 0.000 |
| Planning total score \|9 | -0.638 | 0.131 | -4.865 | 0.000 |
| Planning total score \|10 | -0.448 | 0.126 | -3.547 | 0.000 |
| Planning total score \|11 | -0.422 | 0.126 | -3.357 | 0.001 |
| Planning total score \|12 | -0.322 | 0.124 | -2.593 | 0.010 |
| Planning total score \|13 | -0.153 | 0.122 | -1.250 | 0.211 |
| Planning total score \|14 | -0.082 | 0.122 | -0.673 | 0.501 |
| Planning total score \|15 | 0.082 | 0.122 | 0.673 | 0.501 |
| Planning total score \|16 | 0.397 | 0.125 | 3.167 | 0.002 |
| Planning total score \|17 | 0.822 | 0.138 | 5.958 | 0.000 |
| Abstract logical thinking total score \|1 | -0.999 | 0.147 | -6.815 | 0.000 |
| Abstract logical thinking total score \|2 | -0.322 | 0.124 | -2.593 | 0.010 |
| Abstract logical thinking total score \|3 | 0.129 | 0.122 | 1.058 | 0.290 |
| Abstract logical thinking total score \|4 | 0.527 | 0.128 | 4.115 | 0.000 |
| Abstract logical thinking total score \|5 | 0.889 | 0.141 | 6.309 | 0.000 |
| Abstract logical thinking total score \|6 | 1.441 | 0.181 | 7.967 | 0.000 |
| Visual perception total score \|1 | -1.215 | 0.161 | -7.559 | 0.000 |
| Visual perception total score \|2 | -0.889 | 0.141 | -6.309 | 0.000 |
| Visual perception total score \|3 | -0.667 | 0.132 | -5.050 | 0.000 |
| Visual perception total score \|4 | -0.322 | 0.124 | -2.593 | 0.010 |
| Visual perception total score \|5 | -0.082 | 0.122 | -0.673 | 0.501 |
| Visual perception total score \|6 | 0.371 | 0.125 | 2.976 | 0.003 |
| Visual perception total score \|7 | 0.889 | 0.141 | 6.309 | 0.000 |

Domain level

1. Orientation: The estimation of a one-factor model with eight variables (items in domain “Orientation”) loading on one factor (“Orientation”) failed, which is why we do not report the thresholds for this model.

2. Language: the thresholds for the one-factor model with six variables (items in domain “Language”) that load onto one factor (“Language”)

| Threshold | Estimate | Standard error | z-value | p |
| --- | --- | --- | --- | --- |
| 2.1\|1 | -0.082 | 0.122 | -0.673 | 0.501 |
| 2.1\|2 | 0.012 | 0.122 | 0.096 | 0.923 |
| 2.1\|3 | 0.273 | 0.123 | 2.210 | 0.027 |
| 2.2\|1 | 0.667 | 0.132 | 5.050 | 0.000 |
| 2.3\|1 | -1.038 | 0.149 | -6.976 | 0.000 |
| 2.4\|1 | -0.961 | 0.145 | -6.650 | 0.000 |
| 2.5\|1 | -0.371 | 0.125 | -2.976 | 0.003 |
| 2.5\|2 | -0.035 | 0.122 | -0.289 | 0.773 |
| 2.5\|3 | 0.200 | 0.123 | 1.635 | 0.102 |
| 2.6\|1 | -0.789 | 0.137 | -5.780 | 0.000 |
| 2.7\|2 | -0.554 | 0.129 | -4.304 | 0.000 |
| 2.8\|3 | 0.035 | 0.122 | 0.289 | 0.773 |

3. Attention: the thresholds for the one-factor model with five variables (items in domain “Attention”) that load onto one factor (“Attention”)

| Threshold | Estimate | Standard error | z-value | p |
| --- | --- | --- | --- | --- |
| 3.1\|1 | -1.910 | 0.249 | -7.664 | 0.000 |
| 3.2\|1 | -0.855 | 0.139 | -6.135 | 0.000 |
| 3.3\|1 | -0.474 | 0.127 | -3.737 | 0.000 |
| 3.4\|1 | -0.789 | 0.137 | -5.780 | 0.000 |
| 3.4\|2 | -0.610 | 0.130 | -4.678 | 0.000 |
| 3.4\|3 | -0.422 | 0.126 | -3.357 | 0.001 |
| 3.5\|1 | -0.667 | 0.132 | -5.050 | 0.000 |
| 3.5\|2 | 0.129 | 0.122 | 1.058 | 0.290 |
| 3.5\|3 | 1.378 | 0.175 | 7.891 | 0.000 |

4.Memory: the thresholds for the one-factor model with four variables (items in domain “Memory”) that load onto one factor (“Memory”)

| Threshold | Estimate | Standard error | z-value | p |
| --- | --- | --- | --- | --- |
| 4.1\|1 | -0.667 | 0.132 | -5.050 | 0.000 |
| 4.1\|2 | -0.397 | 0.125 | -3.167 | 0.002 |
| 4.1\|3 | 0.082 | 0.122 | 0.673 | 0.501 |
| 4.2\|1 | -0.500 | 0.127 | -3.926 | 0.000 |
| 4.2\|2 | -0.422 | 0.126 | -3.357 | 0.001 |
| 4.2\|3 | -0.322 | 0.124 | -2.593 | 0.010 |
| 4.2\|4 | -0.153 | 0.122 | -1.250 | 0.211 |
| 4.2\|5 | -0.059 | 0.122 | -0.481 | 0.630 |
| 4.3\|1 | -0.638 | 0.131 | -4.865 | 0.000 |
| 4.3\|2 | -0.346 | 0.124 | -2.785 | 0.005 |
| 4.3\|3 | 0.106 | 0.122 | 0.866 | 0.387 |
| 4.4\|1 | -0.200 | 0.123 | -1.635 | 0.102 |

5. Planning: the thresholds for the one-factor model with nine variables (items in domain “Planning”) that load onto one factor (“Planning”)

| Threshold | Estimate | Standard error | z-value | p |
| --- | --- | --- | --- | --- |
| 5.1\|1 | -1.910 | 0.249 | -7.664 | 0.000 |
| 5.2\|1 | -1.677 | 0.210 | -7.995 | 0.000 |
| 5.3\|1 | -0.697 | 0.133 | -5.234 | 0.000 |
| 5.4\|1 | -0.758 | 0.135 | -5.599 | 0.000 |
| 5.4 \|2 | 0.177 | 0.122 | 1.443 | 0.149 |
| 5.5\|1 | -1.038 | 0.149 | -6.976 | 0.000 |
| 5.5\|2 | -0.297 | 0.124 | -2.402 | 0.016 |
| 5.5\|3 | 0.346 | 0.124 | 2.785 | 0.005 |
| 5.6\|1 | -1.782 | 0.226 | -7.887 | 0.000 |
| 5.6\|2 | -1.677 | 0.210 | -7.995 | 0.000 |
| 5.6\|3 | -0.822 | 0.138 | -5.958 | 0.000 |
| 5.6\|4 | -0.500 | 0.127 | -3.926 | 0.000 |
| 5.7\|1 | -1.215 | 0.161 | -7.559 | 0.000 |
| 5.7\|2 | -1.079 | 0.151 | -7.132 | 0.000 |
| 5.7\|3 | -0.474 | 0.127 | -3.737 | 0.000 |
| 5.7\|4 | -0.397 | 0.125 | -3.167 | 0.002 |
| 5.7\|5 | -0.177 | 0.122 | -1.443 | 0.149 |
| 5.7\|6 | -0.059 | 0.122 | -0.481 | 0.630 |
| 5.8\|1 | -0.758 | 0.135 | -5.599 | 0.000 |
| 5.9\|1 | -0.889 | 0.141 | -6.309 | 0.000 |

6. Abstract-logical thinking: the thresholds for the one-factor model with five variables (items in domain “Abstract-logical thinking”) that load onto one factor (“Abstract-logical thinking”)

| Threshold | Estimate | Standard error | z-value | p |
| --- | --- | --- | --- | --- |
| 6.1\|1 | -0.422 | 0.126 | -3.357 | 0.001 |
| 6.2\|1 | -0.153 | 0.122 | -1.250 | 0.211 |
| 6.2\|2 | 0.322 | 0.124 | 2.593 | 0.010 |
| 6.3\|1 | 0.153 | 0.122 | 1.250 | 0.211 |
| 6.4\|1 | 0.500 | 0.127 | 3.926 | 0.000 |
| 6.5\|1 | 1.122 | 0.154 | 7.282 | 0.000 |

7.Visual perception: the thresholds for the one-factor model with six variables (items in domain “Visual perception”) that load onto one factor (“Visual perception”)

| Threshold | Estimate | Standard error | z-value | p |
| --- | --- | --- | --- | --- |
| 7.1\|1 | -0.610 | 0.130 | -4.678 | 0.000 |
| 7.2\|1 | -0.224 | 0.123 | -1.827 | 0.068 |
| 7.3\|1 | 0.012 | 0.122 | 0.096 | 0.923 |
| 7.3\|2 | 0.727 | 0.134 | 5.418 | 0.000 |
| 7.4\|1 | -0.500 | 0.127 | -3.926 | 0.000 |
| 7.5\|1 | -0.397 | 0.125 | -3.167 | 0.002 |
| 7.6\|1 | -0.697 | 0.133 | -5.234 | 0.000 |
